# ICU patient's body mass index and the critical care outcome: morbidity and mortality

**DOI:** 10.1186/2197-425X-3-S1-A971

**Published:** 2015-10-01

**Authors:** LA Gorordo-Delsol, MTA García-Román, BI Tercero-Guevara, A Jiménez-Ruiz, JA Castañón-González, GD Hernández-López, SE Zamora-Gómez, AI Cuellar-Vasquez, IN Hinojosa-Aguilar, OR Pérez-Nieto, KP Pozos-Cortes

**Affiliations:** Hospital Juárez de México, Unidad de Cuidados Intensivos, Distrito Federal, Mexico

## Introduction

The results of ENSANUT 2012 showed that in a sample of 38208 adults showed 32.4% obesity, besides the increase of 1.3% annually from 2000 through 2012; in the US is reported 66-75% of overweight and obesity in ICU patients; recent studies have shown that obesity, paradoxically, a protective factor in patients admitted to the ICU for surgical pathology.

## Objectives

To report the prevalence of overweight and obesity in the ICU of Hospital Juárez of Mexico (HJM), analyzing the correlation of BMI with AKI, ARDS, LOS

## Methods

254 cases of ICU January 1 to December 31, 2013 were reviewed, demographic variables, height, weight ingress and egress, ingress and egress BMI, admission diagnosis, APACHE II, SOFA recorded , TISS, presence of acute kidney injury (AKI), acute respiratory failure (ARDS), mechanical ventilatory support days (MVS), length of stay in ICU and reason for ICU discharge. The correlation between baseline BMI and the prevalence of AKI, SIRA, AMV days, length of stay in ICU and mortality were analyzed using descriptive statistics.

## Results

The total of 254 records of patients admitted during 2013 were reviewed, data of 108 cases were obtained, were organized into 6 groups according to BMI < 20 (n = 5, 4.62%), 20 to 24.9 (n = 20, 18.51%), 25 to 29.9 (n = 43, 39.81%) 30 34.9 (n = 20, 18.51%) 35 39.9 (n = 14, 12.96%) and> 40 (n = 6, 5.55%) of which 60 were women and 48 men; according to the above groups MVS days were 16, 9.5, 7.48, 17.2, 17.7 and 21.5; the length of stay in ICU: 24.4, 12.1, 12.5, 19.15, 20.5, 23.1; survival in the 6 groups was 80%, 90%, 86%, 85%, 78%, 83%; APACHE II scores averaged joint was 19.8, 14.8, 16, 20.1, 22.1, 23.8, whereas SOFA: 7.8, 6.85, 7.9, 9.35, 10.7, 11.5; and TISS average was 35.6, 35.1, 32, 28.5, 34.9, 42.

## Conclusions

Each BMI has few patients to compare the data in an appropriate way, however, can observe the similarity between BMI of 20 to 24.9 and 25 to 29.9 kg / m2, having duration of MVS, comparable ICU stay; survival was higher in the group of normal BMI over all other groups, with lower APACHE II and SOFA estimated income. The statistic UCIA the HJM is similar to that reported in the international literature, however, no subgroups of patients according to diagnosis were analyzed, so we can not corroborate the "paradox of obese surgical patient". Interesting correlation between BMI, AKI, ARDS and LOS.

## Grant Acknowledgment

The authors do not have any grant to declare.

## References

[1] Akinnusi ME, Pineda LA, El-Solh AA (2008). Effect of obesity on intensive care morbidity and mortality: a meta-analysis. Crit Care Med.

[2] Moock M (2010). Impact of obesity on critical care treatment in adult patients. Rev Bras Ter Intensiva.

[3] Abhyankar S, Leishear K, Callaghan FM, Demmer-Fushman D, McDonald CJ (2012). Lower short- and long-term mortality associated with overeight and obesity in a large cohort study of adult intensive care unit patients. Critical Care.

